# Immersive gratifications and compulsive VR media engagement: a dual-process model of erotic content consumption in virtual reality

**DOI:** 10.3389/fpsyg.2026.1837501

**Published:** 2026-07-02

**Authors:** Intikhab Ahmad, Shahbaz Aslam, Faiz Ullah

**Affiliations:** 1 School of Journalism and Communication, Anhui Normal University, Wuhu, Anhui, China; 2 Department of Media Studies, Bahria University Islamabad Campus, Islamabad, Pakistan; 3 Centre for Media and Communication Studies, University of Gujrat, Gujrat, Pakistan; 4 School of Media Studies, Superior University, Lahore, Pakistan

**Keywords:** compulsive engagement, dual-process theory, sense of presence, uses and gratifications, virtual reality

## Abstract

**Introduction:**

Drawing on dual-process theory and uses-and-gratifications (U&G) perspectives, this study develops the Immersive Gratification Model (IGM) to explain compulsive engagement with erotic/pornographic content (EPC) in virtual reality (VR).

**Methods:**

The IGM distinguishes three distal gratification-seeking predictors: sensual curiosity (SC), VR self-efficacy (VSE), and excitement-seeking and EPC pleasure (ESEPCP) from two proximal immersive experience evaluations: VR sense of presence (VRP), representing an automatic perceptual pathway, and VR cognitive satisfaction (VCS), representing a deliberate evaluative pathway. Both proximal evaluations are theorized to predict compulsive VR-EPC engagement (VRA), with habit (Hbt) as an asymmetric boundary-condition moderator. Survey data from 388 adult VR-EPC consumers (aged 18+) were analyzed using partial least squares structural equation modelling.

**Results:**

All three distal predictors are positively associated with both VRP and VCS, which in turn predict VRA. Mediation analyses support sequential indirect effects through both pathways. Notably, habit amplifies the VRP → VRA path but not the self-sustaining VCS → VRA path, revealing an asymmetric dual-process moderation pattern.

**Discussion:**

These findings extend U&G theory to immersive VR media, advance dual-process accounts of problematic technology use, and carry implications for VR platform design and digital well-being intervention.

## 1 Introduction

The arrival of consumer-grade VR headsets has introduced a qualitatively new mode of erotic/pornographic content (EPC) consumption, one that is immersive, embodied, and increasingly indistinguishable from physical presence (Bojić et al., 2026; Elsey et al., 2019). Unlike passive, screen-based viewing, VR-based EPC engages proprioceptive, spatial, and attentional systems simultaneously, raising the question of whether established theoretical accounts of problematic media use adequately explain engagement patterns in fully immersive environments.

In this study, we integrate dual-process theory (System 1/automatic vs. System 2/deliberate processing) with uses-and-gratifications (U&G) perspectives to explain compulsive VR EPC engagement. We propose that distal gratification-seeking antecedents are positively associated with two proximal immersive-experience evaluations, VR sense of presence (VRP) and VR cognitive satisfaction (VCS), which in turn are positively associated with virtual reality erotic/pornographic content engagement addiction (VRA). Habit (Hbt) is theorized as a moderating boundary condition that selectively amplifies automatic (presence-driven) but not deliberate (satisfaction-driven) pathways to compulsive use, yielding an asymmetric dual-process prediction.

Importantly, although the study sample is limited to VR-based EPC consumers, the findings offer tentative implications for VR content creators and platform designers. Broader generalizations to other VR industries should not be made without further empirical validation in those contexts. This study makes three contributions to the literature. First, it introduces an immersive gratification model that extends U&G theory into VR-specific contexts, demonstrating that the gratification architecture of immersive media creates distinct pathways to compulsive engagement. Second, it advances dual-process accounts of problematic technology use by providing the first empirical test of asymmetric habit moderation on presence- versus satisfaction-driven addiction pathways in VR. Third, it addresses important gaps in the VR media psychology literature by examining an understudied but rapidly growing content domain. Knowing that compulsive media behaviors have a deteriorating effect on the health and psychological wellbeing of consumers, the study’s practical implications will focus on evidence-based insights for VR platform designers, digital wellbeing researchers, and media literacy educators.

## 2 Literature review

### 2.1 Virtual reality, erotic content, and the research gap

Virtual reality (VR) places users inside an interactive simulation, generating a compelling sense of physical presence rather than merely observing a screen. Its defining affordances, embodied immersion, spatial interactivity, and real-time customization produce psychological effects qualitatively distinct from flat-screen media, driving rapid adoption across education, healthcare, and entertainment (Maples-Keller et al., 2017; Sung et al., 2024). The pornography industry adopted VR particularly quickly, and user surveys now document rising consumption of VR-based erotic/pornographic content (EPC) alongside emerging reports of loss of control (Elsey et al., 2019; Shu et al., 2025).

EPC refers to professionally or non-professionally produced sexualized media intended to elicit sexual arousal (Peter and Valkenburg, 2016). Its 2D screen-based form has been studied extensively: research consistently finds that while EPC can serve legitimate functions of sexual exploration and stress relief, it is also linked to compulsive use, relationship dissatisfaction, and psychological distress (Daspe et al., 2018; Privara and Bob, 2023). Male and younger users face disproportionately elevated compulsive risk (Ballester-Arnal et al., 2023). Crucially, virtually all empirical work on problematic EPC concerns 2D delivery formats. How the embodied, immersive properties of VR alter the gratification-to-compulsion dynamic remains poorly understood, and the present study is designed to fill this gap.

### 2.2 Theoretical foundations

The Immersive Gratification Model (IGM) integrates two complementary theoretical traditions. Uses-and-gratifications (U&G) theory holds that audiences actively select media to satisfy specific psychological needs, distinguishing gratifications sought, the motivational orientations brought to a media encounter, from gratifications obtained, and the rewards actually derived (Katz et al., 1973; Rubin, 2009). Extensions of U&G to interactive platforms show that medium-specific affordances create entirely new gratification categories beyond those available in broadcast media (Sundar and Limperos, 2013). For VR, the presence affordance is one such novel category: a medium-level gratification that intensifies engagement independently of content. The IGM treats VR sense of presence (VRP) and VR cognitive satisfaction (VCS) as VR-specific gratifications obtained that mediate between users’ motivational dispositions and compulsive re-engagement.

Dual-process theory distinguishes automatic (System 1) processes—fast, associative, and effortless—from deliberate (System 2) processes, slow, rule-governed, reflective (Evans and Stanovich, 2013; Strack and Deutsch, 2004). In technology compulsion, automatic processing underlies cue-triggered approach behavior while deliberate processing governs evaluative judgments about experience value. The IGM maps this onto VRP, which is a System-1-adjacent automatic perceptual response arising pre-reflectively from the immersive environment, and VCS, which is a System-2-adjacent deliberate post-episode appraisal. These are theoretical analogies, not direct operationalizations, employed to ground the model’s central asymmetric moderation prediction.

### 2.3 Predictors

Distal predictors are stable motivational and capability-based characteristics determining what gratifications users seek before a VR-EPC episode begins. Three are theorized: sensual curiosity (SC), VR self-efficacy (VSE), and excitement-seeking and EPC pleasure (ESEPCP). Each is grounded in established literature and is expected to shape the proximal experiences of presence and cognitive satisfaction that, in turn, drive compulsive engagement.

Sensual curiosity (SC) is a stable tendency to seek novel erotic experiences. Curiosity is activated when individuals perceive a knowledge gap and are motivated to close it (Loewenstein, 1994). VR’s 360° environments amplify erotic curiosity by presenting genuinely novel stimuli relative to 2D formats (Cen et al., 2024), and prior research on 2D EPC consumption shows that curiosity-driven access predicts sustained and escalating engagement (Foubert et al., 2011). Users high in SC approach, VR-EPC with heightened attention and openness, generate stronger felt presence and greater post-episode cognitive satisfaction.

*H1a*: Sensual curiosity (SC) is positively associated with VR sense of presence (VRP).

*H1b*: Sensual curiosity (SC) is positively associated with VR cognitive satisfaction (VCS).

The VR self-efficacy (VSE) captures users’ confidence in operating VR systems. Higher self-efficacy produces deeper engagement and richer experiential outcomes (Bandura, 1978; Castronovo et al., 2024). In VR-EPC contexts, users who feel competent at navigating the headset and customizing scenarios are better positioned to generate high-presence sessions experienced as cognitively satisfying. This reframes self-efficacy from an adoption facilitator to an enabler of compulsive re-engagement: competence at VR-EPC consumption increases the likelihood that each episode delivers the gratifications motivating the next.

*H2a*: VR self-efficacy (VSE) is positively associated with VRP.

*H2b*: VR self-efficacy (VSE) is positively associated with VCS.

Excitement-seeking and EPC pleasure (ESEPCP) reflects a hedonic orientation toward intense, arousal-rich erotic experiences. Highly pleasurable activities generate their own motivational momentum: the pleasure of the experience itself drives re-engagement (Cziksentmihalyi, 1990; Fang, 2024). VR amplifies such states because multisensory stimulation and embodied presence intensify emotional arousal well beyond what 2D formats deliver (Elsey et al., 2019; Lønne et al., 2023). Users high in ESEPCP attend deeply to VR-EPC episodes, translating into stronger presence and greater post-episode satisfaction.

*H3a*: Excitement-seeking and EPC pleasure (ESEPCP) is positively associated with VRP.

*H3b*: Excitement-seeking and EPC pleasure (ESEPCP) is positively associated with VCS.

### 2.4 Proximal evaluations and compulsive engagement

VR sense of presence (VRP) is the subjective sense of ‘being in’ the simulated environment rather than observing it from the outside (Schubert et al., 2001; Slater, 1999). Presence amplifies emotional responses, heightens behavioral intentions, and makes simulated scenarios feel personally relevant (Austermann et al., 2025; Hameed and Perkis, 2024). In EPC contexts, high presence minimizes real-world distractions and amplifies sensory involvement, generating intensity unavailable in 2D formats (Lønne et al., 2023). The mechanism linking VRP to compulsive re-engagement is automatic: the memory of the presence experience functions as a salient contextual cue that triggers approach behavior without deliberate decision.

*H4a*: VR sense of presence (VRP) is positively associated with compulsive VR-EPC engagement (VRA).

The VR cognitive satisfaction (VCS) is the deliberate evaluative judgment that a completed VR-EPC episode has met or exceeded expectations (Caro and García, 2007; Mano and Oliver, 1993). In media and service research, cognitive satisfaction consistently predicts continued and escalating use by creating positive outcome expectations that justify future engagement (Amoroso and Lim, 2017; Perry, 2016). The IGM positions VCS as a deliberate gratification-obtained evaluation that feeds back into compulsive use through a reasoning-based reinforcement pathway. Since both VRP and VCS transmit the effects of the distal predictors onto VRA, the model implies six indirect effects.

*H4b*: VR cognitive satisfaction (VCS) is positively associated with compulsive VR-EPC engagement (VRA).

*H5*: VRP mediates the SC → VRA (H5a), VSE → VRA (H5b), and ESEPCP → VRA (H5c) associations.

*H6*: VCS mediates the SC → VRA (H6a), VSE → VRA (H6b), and ESEPCP → VRA (H6c) associations.

### 2.5 The moderating role of habit

Habit refers to context-triggered automatic behavior performed with little conscious deliberation (Gardner et al., 2024; Stojanovic et al., 2022). In information systems research, it is among the strongest predictors of continued technology use (Jeyaraj, 2022), and in EPC research, it explains entrenched viewing patterns and the progression toward compulsive use (Tülübaş et al., 2023). The IGM does not treat habit as a uniform amplifier of continued engagement. Instead, it proposes that habit operates as an asymmetric moderator whose effect depends on whether the proximal pathway it acts upon is automatic or deliberate.

For the VRP → VRA path, habit and presence are congruent in their automaticity. Repeated high-presence VR-EPC sessions condition cue-triggered re-engagement, so at higher habit levels, the VR context alone initiates approach behavior without deliberation. For the VCS → VRA path, habit adds nothing: cognitive satisfaction already functions as a self-sustaining deliberate reward signal that motivates return visits through positive outcome expectation, a reasoning process operating independently of habitual automaticity. Introducing habit to this pathway is theoretically redundant, and no moderating effect is predicted. This asymmetric prediction is the IGM’s most distinctive theoretical contribution.

*H7a*: Habit (Hbt) positively moderates the VRP → VRA association such that the relationship is significantly stronger at higher levels of habit.

*H7b* was reformulated during revision following reviewer feedback to more precisely reflect dual-process theory; the original moderation hypothesis has been retained, and this revision is explicitly disclosed.

*H7b*: The data did not provide evidence that habit significantly moderated the VCS → VRA association; equivalence or Bayesian methods would be needed to establish the absence of moderation.

**Figure 1:**
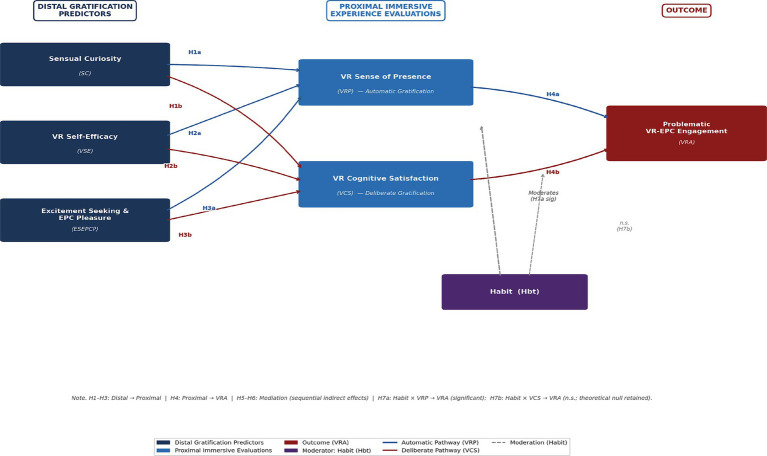
Conceptual model.

This is interpreted as consistent with the theoretical prediction that cognitive satisfaction constitutes a self-sustaining deliberate reward signal that does not require habitual priming; however, this interpretation should be treated with caution, given that non-significance does not establish evidence of absence (Figure 1).

## 3 Method

Methodologically, the authors requested that VR-based EPC consumers participate in the electronic questionnaire survey. To access the potential sample candidates, the VR-based EPC consumers use social networking sites (SNS) (specifically Facebook). The related pages, which include comments, reviews, and updates on VR-based EPC, are targeted. Prior related behavioral studies also considered the mentioned SNS as a typical tool. This study involved a questionnaire-based survey of 388 adult human participants (aged 18 years and above). Ethical approval was obtained from the Institutional Review Board of the University (IRB Protocol No. ANU-IRB-2024-A75). All participants were informed of the study’s purpose, their right to withdraw at any time without penalty, and the confidentiality of their responses. Written informed consent was obtained electronically from all participants before commencing the survey. No deception was used, and no sensitive personal data beyond anonymous self-reports was collected.

### 3.1 Instrument design

All constructs were measured using multi-item 5-point Likert scales (1 = strongly disagree, 5 = strongly agree) adapted from prior validated instruments. Sensual curiosity (SC; 4 items) and excitement seeking and EPC pleasure (ESEPCP; 3 items) were adapted from Kim et al. (2018). VR self-efficacy (VSE; 3 items) was adapted from Suki and Ramayah (2010). VR presence (VRP; 3 items) was measured using items from Bogicevic et al. (2019), and VR cognitive satisfaction (VCS; 3 items) was adapted from Kim et al. (2018). The scale of Habit (Hbt; 3 items) was measured through items from Pillet and Carillo (2016). The VR experience of addiction to EPC (VRA; 3 items) was assessed using an adapted version of the mobile social networking addiction scale by Gong et al. (2020), which includes the respondents’ perceived loss of control and compulsive use of VR EPC. Following previous research using short dependency instruments, VRA is considered a marker of problematic overuse and not as a clinical diagnosis.

The adapted items were reviewed by 10 experts (four academic researchers and six graduate students who are fluent in English as volunteers). A few minor wording changes were made in accordance with their suggestions, but there were no items deleted. The survey was not conducted in a language other than English, and the survey population was not in any language other than English, so formal translation-back-translation was not necessary. VR EPC addiction (VRA) was measured by a three-item scale that measures perceived loss of control and compulsive use of EPCs through VR. As with previous research on behavioral dependence with short, unidimensional scales, we do not view VRA as a clinical addiction, but as a tendency to problematic overuse. Items for sensual curiosity (SC; four items) and excitement seeking and EPC pleasure (ESEPCP; four items) were originally designed by Kim et al. (2018) for the context of switching between mobile phones and later adapted to the EPC/pornography context by Leon-Larios et al. (2019).

Lawshe’s (1975) content validity framework was used for justifying the expert panel, which included both domain experts and knowledgeable respondents. The four academic researchers (faculty with publications in VR, media psychology, and behavioral addiction) assessed theoretical relevance and construct alignment. The six PhD candidates, all with completed coursework in psychometric measurement, research methods, and VR technology, assessed item clarity, readability, and face validity from a respondent-proxy perspective. This mixed-expertise panel is consistent with recommendations for content validity assessment in sensitive-topic survey research (Lynn, 1986). Construct Clarification: The VRA scale captures self-reported compulsive engagement tendencies, including uncontrolled use, preoccupation, and loss of control, rather than formal clinical addiction. Throughout this manuscript, ‘problematic VR-EPC engagement’ is the preferred term, while ‘VRA’ is retained as the abbreviation for the measured construct for consistency with the path model.

No formal quantitative content validity ratio (CVR) or content validity index (CVI/S-CVI) values were calculated. The expert review process should therefore be characterized as a structured qualitative expert review rather than a formal quantitative content-validation procedure in the Lawshe (1975) or Lynn (1986) sense. Reviewer feedback was used to assess item clarity and theoretical alignment; minor wording changes were incorporated based on consensus.

### 3.2 Collection and description

First-hand data were collected from VR-based erotic/pornography content (EPC) consumers reached through related web resources, particularly Facebook, which hosts the social networking pages of major VR-based EPC providers. We employed non-probability convenience sampling from these pages. We identified users who had interacted with VR-based EPC pages and whose privacy settings allowed them to receive friend requests. We then contacted these users individually via private message, briefly introduced the study, and shared the survey cover and link. Contact with potential participants was initiated only through standard platform functions and in accordance with Facebook’s terms of use. We did not preselect potential respondents based on demographic characteristics (e.g., gender) or visual judgments of profile pictures. The only inclusion criteria were as follows: (1) willingness to receive our friend request and message, (2) self-reported prior experience of consuming VR-based EPC, and (3) aged 18 years or older. The inclusion criterion required prior experience of consuming VR-based EPC content, aged 18 years or older, but did not require current VR device ownership. Respondents who did not personally own a VR device may have accessed VR-based EPC through a friend’s, partner’s, or venue-based device. This approach is consistent with prior VR research that distinguishes between device ownership and VR use experience (Herz and Rauschnabel, 2019). Table 1 reflects this distinction; respondents without personal VR ownership had still experienced VR-based EPC, which was the qualifying criterion for inclusion.

**Table 1:** Surveyed sample profile.

Characteristic	Detail	Frequency	Percentage
Gender	Male	325	83.76
	Female	63	16.24
Age	Under 25	227	58.50
	25–35	98	25.26
	Above 35	63	16.24
Being in a relationship?	Yes	142	36.60
	No	246	63.40
Having VR device/gadget?	Yes	319	82.22
	No	69	17.78
Education	Attended School	74	19.07
	Attended College (include vocational)	231	59.54
	Attended University	83	21.39

Given the sensitive nature of pornography-related behaviors, all data were collected through a self-administered online questionnaire. Respondents completed the survey in their own time and on their own devices, and we did not ask for personally identifying information (e.g., real names or contact details). Participants were assured that their responses would be treated confidentially, would be analyzed only in aggregate form, and that there were no “right” or “wrong” answers. These procedures were intended to reduce social desirability pressures and encourage honest reporting, although we acknowledge that self-report remains imperfect for highly sensitive topics.

Data collection took approximately 7 months (15 March 2024–15 October 2024). To encourage participation, respondents were informed that survey completion would enter them into a cryptocurrency prize draw (12,000–120,000 Satoshi; approximately USD 1–10). This incentive is acknowledged as a potential source of response bias. Of 437 initial responses, 49 were excluded due to incomplete responses (*n* = 31), failed attention-check items (*n* = 12), and evidence of straight-lining (*n* = 6), yielding 388 valid cases. Following Armstrong and Overton (1977), early and late respondents were compared via chi-square tests; no significant differences were found, indicating that non-response bias is unlikely to be a major concern. The sample is predominantly male (83.76%), young, and relatively well-educated (see Table 1). Participants were recruited from Facebook pages dedicated to VR-based EPC providers, which likely overrepresents habitual, high-frequency consumers; findings should therefore be interpreted as characterizing relationships within an active-user subgroup and should not be generalized to the broader VR-using population without caution.

## 4 Results

The results comprise three sections: measurement model evaluation, structural analysis, mediation and moderation testing, as proposed in Section 2.

### 4.1 Measurement analysis

The internal and external reliability and validity were measured through Exploratory and Confirmatory Factor Analysis (E&CFA). Cronbach’s alpha (*α*) for each construct was in the range of 0.797–0.946. Average variance extracted (AVE) was recorded within the continuum of 0.567 and 0.897, as shown in Table 2. Composite reliability (CR) was observed within the band of 0.797 and 0.963. Specifically, all values of α, AVE, and CR witnessed within the acceptable ranges, as suggested by Hair et al. (2010), to classify the adapted instruments as valid. All standardized factor loadings were above 0.733 (see Table 2), exceeding the recommended minimum threshold of 0.708 for indicator reliability (Hair et al., 2010).

**Table 2:** Exploring factors and reliability analysis.

Construct	Items	λ	*α*	CR	AVE
Sensual Curiosity (SC)	SC1	0.879	0.901	0.906	0.707
	SC2	0.863			
	SC3	0.836			
	SC4	0.781			
Excitement Seeking and EPC Pleasure (ESEPCP)	ESEPCP1	0.832	0.776	0.823	0.610
	ESEPCP2	0.773			
	ESEPCP3	0.735			
Virtual Self-Efficacy (VSE)	VSE1	0.788	0.750	0.797	0.567
	VSE2	0.736			
	VSE3	0.733			
VR Presence (VRP)	VRP1	0.892	0.924	0.903	0.757
	VRP2	0.871			
	VRP3	0.848			
VR Cognitive Satisfaction (VCS)	VCS1	0.847	0.891	0.872	0.698
	VCS2	0.838			
	VCS3	0.822			
VR EPC Addiction (VRA)	VRA1	0.819	0.814	0.820	0.603
	VRA2	0.773			
	VRA3	0.735			
Habit (Hbt)	Hbt1	0.959	0.946	0.963	0.897
	Hbt2	0.943			
	Hbt3	0.940			

The analysis proceeded in three stages. First, an exploratory factor analysis (EFA) was conducted on a randomly split holdout subsample (*n* = 50) to assess the dimensionality of adapted items and identify any cross-loading items; factors were retained using the eigenvalue > 1 criterion and parallel analysis. Second, confirmatory factor analysis (CFA) was conducted on the full sample (*n* = 388) within a partial least squares SEM (PLS-SEM) framework implemented in SmartPLS 4.0 using the consistent PLS (PLSc) algorithm appropriate for reflective constructs. Items with outer loadings below 0.70 were candidates for removal; all retained items met this threshold (see Table 2). Missing data were minimal (<1% of cells) and handled via listwise deletion. Normality was assessed using Mardia’s multivariate normality test; given violations, all significance tests were conducted via bootstrapping with 5,000 resamples, producing bias-corrected confidence intervals. Common method bias was assessed using Harman’s (1976) single-factor test; no single factor explained the majority of variance, suggesting CMB is not a serious concern. The EFA holdout sample (*n* = 50) is admittedly small; however, given that all scales were adapted from validated instruments and used a limited number of items (4–5 per construct), a small holdout EFA serves primarily as a screening check rather than a standalone factor analysis. All items loaded cleanly (> 0.50) on their intended factors with no cross-loadings (<0.35), providing adequate confidence that scale adaptation did not introduce structural distortions. Readers who prefer a CFA-only approach may treat the CFA on *n* = 388 as the primary validity evidence.

To assess discriminant validity, we followed the Fornell–Larcker criterion by comparing the square root of each construct’s AVE with its correlations with other constructs (Fornell and Larcker, 1981). As shown in Table 3, for every construct, the square root of the AVE (diagonal) exceeds the corresponding inter-construct correlations, supporting discriminant validity (Hair et al., 2010). In addition, we inspected cross-loadings and confirmed that each indicator loaded more strongly on its intended construct than on any other construct. Finally, multicollinearity among the predictors of VRA was examined via variance inflation factors (VIFs), which ranged from 1.01 to 1.58 (Table 3), well below common cut-offs, indicating that multicollinearity is unlikely to bias the structural estimates. In addition, inspection of the cross-loading pattern showed that each item loaded more strongly on its intended construct than on any other, providing further support for discriminant validity.

**Table 3:** Discriminant reliability and correlation analysis.

Construct	*M*(SD)	VIF	SC	ESEPCP	VSE	VRP	VCS	Hbt	VRA
SC	2.894(0.806)	1.372	**0.840**						
ESEPCP	2.808(0.808)	1.110	0.294	**0.781**					
VSE	3.588(0.649)	1.326	0.299	0.171	**0.752**				
VRP	4.005(0.653)	1.393	0.330	0.220	0.550	**0.870**			
VCS	3.635(0.796)	1.575	0.462	0.245	0.379	0.454	**0.835**		
Hbt	3.763(0.652)	1.011	0.087	0.055	0.019	0.043	0.075	**0.947**	
VRA	3.982(0.548)	–	0.307	0.231	0.423	0.480	0.416	0.062	**0.776**

*p < 0.05, **p < 0.01, ***p < 0.001. Diagonal bold values represent the square root of AVE; off-diagonal values are inter-construct correlations. VIF values are presented in the VIF column.

Common method bias (CMB) was assessed using multiple procedures. First, Harman’s (1976) single-factor test confirmed that no single factor accounted for more than 50% of the variance (largest factor = 31.31%). Second, a full collinearity assessment was conducted: all inner VIF values fell below 3.3 (range: 1.18–2.94), indicating that multicollinearity and CMB are not a concern (Kock, 2015). Third, a single-factor CFA model was estimated; its substantially poorer fit relative to the proposed measurement model (ΔCFI > 0.10; ΔRMSEA > 0.05) confirms that common method variance is unlikely to account for the observed factor structure. These converging results suggest that CMB does not represent a serious threat to the validity of the findings.

### 4.2 Structural analysis

Analytical strategy note: Although this study initially employed a PLS-SEM framework (SmartPLS 4.0, PLSc algorithm), model fit was ultimately evaluated using covariance-based SEM indices via AMOS 24 to provide the conventional fit statistics required by target journals. PLSc was used for path coefficient estimation and bootstrapped confidence intervals; CB-SEM fitness indices are reported for model evaluation purposes only. Both approaches yielded highly consistent structural results.

**Figure 2:**
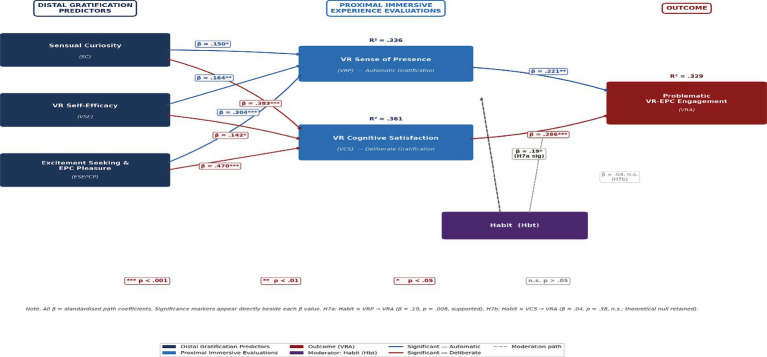
Graphical view of path analysis of the proposed model.

The measurement and structural models both exhibit excellent fit. For the measurement model: *χ*^2^(127) = 171.99, *χ*^2^/df = 1.35; GFI = 0.96, AGFI = 0.93, TLI = 0.99, CFI = 0.99, NFI = 0.96, RMSEA = 0.030. For the structural model: *χ*^2^(126) = 173.55, *χ*^2^/df = 1.38; GFI = 0.96, AGFI = 0.93, TLI = 0.99, CFI = 0.99, NFI = 0.96, RMSEA = 0.031. All indices exceed conventional thresholds, indicating a close fit to the data. All proposed hypotheses were supported at *p* < 0.05, except H7b (habit × VCS moderation), which was non-significant, as discussed below. Standardized path coefficients are presented in Figure 2. ESEPCP was the strongest distal predictor (H3a: *β* = 0.304 on VRP; H3b: *β* = 0.470 on VCS). SC was more influential for VCS (H1b: *β* = 0.383) than for VRP (H1a: *β* = 0.150). VSE showed comparable effects on both proximal constructs (H2a: *β* = 0.164; H2b: *β* = 0.142). VCS was the stronger proximal predictor of VRA (H4b: *β* = 0.286) relative to VRP (H4a: *β* = 0.221). The distal predictors explained 33.6% of the variance in VRP and 36.1% in VCS; the proximal constructs explained 32.9% of the variance in VRA.

### 4.3 Mediation and moderation evaluation

To assess mediation, asymmetric confidence intervals for indirect associations measured through the bootstrap sampling method (with a bootstrap sample size of 5,000) as suggested by MacKinnon et al. (2004). The literature argues that measuring confidence intervals through bootstrap provides more accurate and precise estimations compared to traditional modes and methods, that is, the Sobel test (MacKinnon et al., 2004). Table 4 comprises the outcome of the mediation analysis. The findings conclude that the listed proximal causes mediate all proposed distal causes in the study, as none of the recorded confidence intervals hold ‘zero’ within the lower and upper bounds. In other words, H5 and H6 conclude supported results while testing mediation through bootstrapping.

**Table 4:** Results for the bootstrapping method for mediation.

Hypo	IV	M	DV	Effect of IV on M	Effect of M on DV	Direct (c’)	Indirect (a*b)	Total effect (c)	95% (Cl)	Mediation
H5(a)	SC	VRP	VRA	0.248***	0.245***	0.074*	0.061***	0.224***	(0.034, 0.097)	Supported
H5(b)	VSE	VRP	VRA	0.187***	0.245***	.044 ns	0.046**	0.149***	(0.027, 0.075)	Supported
H5(c)	ESEPCP	VRP	VRA	0.425***	0.161***	0.317***	0.069**	0.464***	(0.030, 0.109)	Supported
H6(a)	SC	VCS	VRA	0.397***	0.224**	0.074*	0.089***	0.224***	(0.047, 0.132)	Supported
H6(b)	VSE	VCS	VRA	0.238***	0.251***	.044 ns	0.060**	0.149***	(0.034, 0.092)	Supported
H6(c)	ESEPCP	VCS	VRA	0.422***	0.188***	0.317***	0.079**	0.464***	(0.042 0.127)	Supported

*** = Significance level of 0.001, ** = Significance level of 0.01, * = Significance level of 0.05.

Mediation type was further assessed by examining whether direct effects remained significant in the saturated model (i.e., after including VRP and VCS as mediators). All distal-to-VRA direct paths were attenuated but remained significant (*p* < 0.05), indicating partial mediation across most pathways. The single exception is the VSE → VCS → VRA pathway, where the direct VSE → VRA path became non-significant upon inclusion of VCS, indicating full mediation for this specific pathway. These results are consistent with the bootstrapping evidence in Tables 4, 5.

**Table 5:** Results for the bootstrapping method for mediation.

Hypo	IV	M	DV	IV → DV	IV → M	IV + M → DV		Mediation
						IV → DV	M → DV	
H5(a)	SC	VRP	VRA	0.224***	0.248***	0.137***	0.351***	Partial
H5(b)	VSE	VRP	VRA	0.149***	0.187***	0.077*	0.380***	Partial
H5(c)	ESEPCP	VRP	VRA	0.465***	0.425***	0.357***	0.253***	Partial
H6(a)	SC	VCS	VRA	0.224***	0.397***	0.086*	0.349***	Partial
H6(b)	VSE	VCS	VRA	0.149***	0.237***	.060 ns	0.377***	Full
H6(c)	ESEPCP	VCS	VRA	0.465***	0.422***	0.355***	0.259***	Partial

***p < 0.001, **p < 0.01, *p < 0.05, ns = not significant.

**Figure 3:**
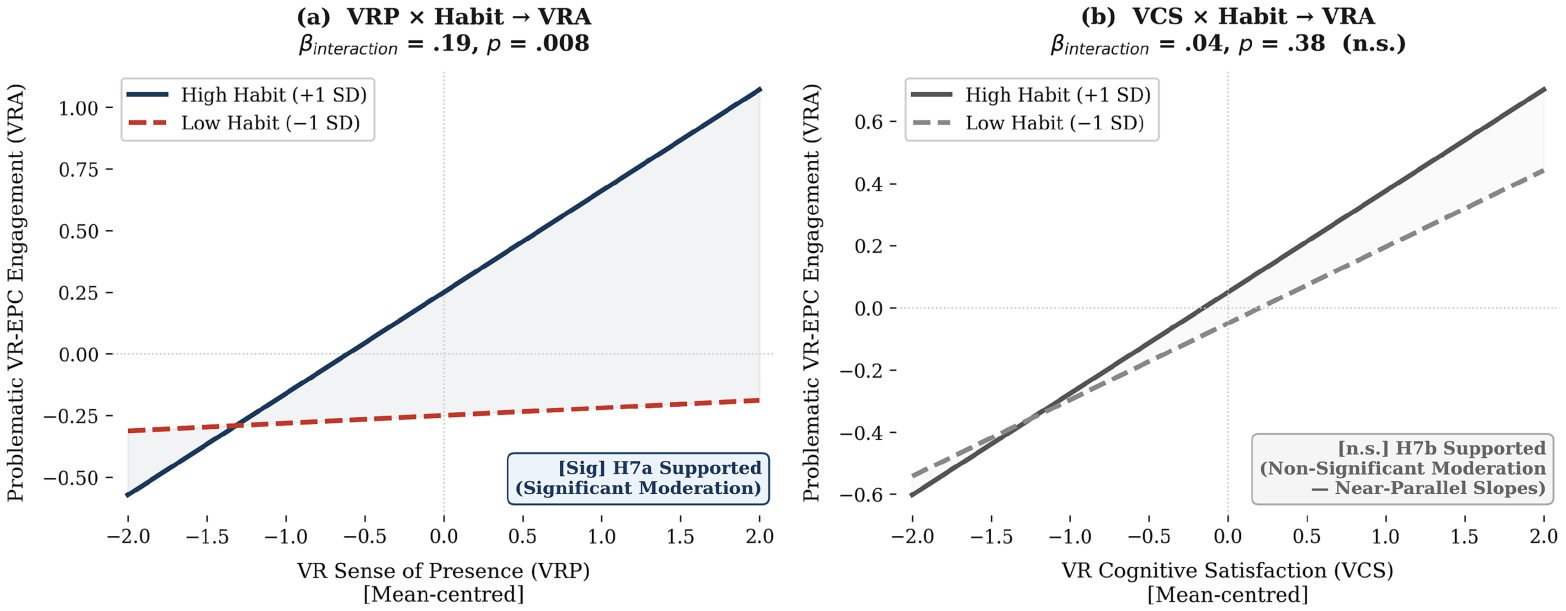
Moderating role of habit (Hbt) on proximal → VRA pathways (asymmetric dual-process moderation). **(a)** VRP × Habit → VRA: significant moderation (β interaction = 0.19, *p* = 0.008), showing a stronger positive association for high-habit than low-habit users. **(b)** VCS × Habit → VRA: non-significant moderation (β interaction = 0.04, *p* = 0.38), with near-parallel slopes indicating absence of meaningful moderation.

To further understand the impact of proximal causes (VRP and VCS) on VRA, the moderating effect of Hbt was measured with the help of hierarchical regression testing. The results highlighted that the presence of Hbt to use VR gadgets while viewing EPC strengthened the association between VRP and VRA (IGM-H7a: *β* = 0.116, *p* ≤ 0.05). However, in the case of VCS, Hbt failed to mark the impact as proposed in IGM-H7b (IGM-H7b: *β* = 0.03, *p* ≥ 0.05), as graphically shown in Figures 3a,b. Figure 3b displays the Hbt × VCS interaction plot for completeness; readers should note that this interaction is non-significant (*p* = 0.38), and the near-parallel slopes indicate the absence of meaningful moderation. The visual similarity of the two slope lines in Figure 3b reflects this non-significance and should not be interpreted as indicating a meaningful interaction effect.

## 5 Discussion

This study tested the immersive gratification model (IGM), an integration of dual-process theory and uses-and-gratifications (U&G) perspectives, in the context of VR-based erotic/pornographic content (EPC) consumption. The findings confirm that all three distal gratification-seeking predictors (SC, VSE, and ESEPCP) are positively associated with both proximal immersive experience evaluations (VRP and VCS), which in turn are positively associated with problematic VR-EPC engagement (VRA). ESEPCP emerged as the most influential distal predictor, consistent with prior evidence that hedonic motives are strong drivers of immersive media engagement (Maples-Keller et al., 2017; Saneinia et al., 2022). Mediation tests confirm the sequential gratification chain, and moderation results reveal the theoretically predicted asymmetry: habit amplifies the automatic (VRP-driven) but not the deliberate (VCS-driven) pathway to problematic engagement, consistent with the dual-process logic of the IGM. These findings advance our understanding of immersive media compulsion and are discussed in the following subsections.

### 5.1 Theoretical implications

The IGM extends U&G theory to immersive VR by demonstrating that VR’s gratification architecture creates pathways from gratification-seeking to compulsive engagement that screen-media U&G models do not capture. Specifically, VR-EPC provides both content gratifications (arousal, curiosity, and pleasure) and a medium gratification (embodied presence) that independently predicts problematic engagement, a distinction with implications for how U&G theory conceptualizes gratifications obtained in immersive contexts. The IGM’s positioning of the CBM within a U&G and dual-process framework resolves a theoretical gap: CBM accounts for foreground maladaptive reinforcement but cannot explain why VR-specific affordances intensify compulsion beyond 2D media.

Second, the IGM provides the first empirical test of asymmetric dual-process habit moderation in immersive media compulsion. Habit amplifies only the automatic (VRP → VRA) pathway, not the deliberate (VCS → VRA) pathway, establishing a novel boundary condition for technology compulsion theory: the pathway-contingent nature of habit’s role. VSE, in the IGM, also functions as an enabler of compulsion rather than merely an adoption facilitator; users high in VR self-efficacy more effectively extract presence-based and satisfaction-based gratifications, increasing their vulnerability to compulsive re-engagement.

Within the IGM, VR self-efficacy (VSE) functions as a capability-enabling gratification predictor: users high in VSE are better equipped to extract both presence-based and satisfaction-based gratifications from VR-EPC episodes, thereby increasing their vulnerability to compulsive re-engagement. Particularly, VCS fully mediates the VSE → VRA path, meaning that VR competence is problematic when it can regularly produce cognitively rewarding EPC experiences, but not when accessing the devices. This pushes the definition of self-efficacy from an IS concept to a concept of enabling compulsion in immersive media environments, building on previous IS research, which studied self-efficacy mostly as a predictor for the adoption of technology. On a practical level, interventions focusing solely on the skills and the access to the device without addressing the cognitive satisfaction of using VR-EPCs are likely to be ineffective.

The IGM’s most notable theoretical contribution is the asymmetric pattern of habit moderation across the VRP → VRA and VCS → VRA routes. Consistent with the reflective-impulsive model (Strack and Deutsch, 2004), habit amplified the automatic VRP → VRA path but did not provide statistically significant evidence of moderating the deliberate VCS → VRA path. This pattern is consistent with the theoretical prediction that deliberate cognitive satisfaction is a self-sustaining reward signal, though it must be noted that the absence of a significant interaction does not, by itself, establish evidence of the absence of moderation; future studies employing equivalence testing or Bayesian methods would be needed to more firmly establish this null result (Altman and Bland, 1995; Dienes, 2014). This finding parallels Brand et al. (2016) dual-process addiction model, in which cue-reactivity (analogous to VRP) is more susceptible to habitual conditioning than outcome valuation (analogous to VCS). Future studies should confirm whether this asymmetry generalizes to other immersive content types and whether habit-disruption interventions differentially attenuate the two pathways.

These theoretical implications suggest that the IGM can be considered a tool beyond simply characterizing immersive media compulsion as a one-dimensional construct, bringing an academically sound basis for pathway-specific intervention and measurement approaches for future research.

### 5.2 Practical implications

The IGM’s conclusions have focused on implications for VR platform designers, digital wellbeing scholars, and media literacy practitioners. Platform designers are therefore faced with the challenge of finding an intervention architecture that applies the dual processes of problematic VR-EPC engagement, which calls for different approaches to the platforms. Presence-driven compulsion is an automatic pathway that is sharpened by habit and is triggered by disruptions in context (e.g., enforced session breaks, environmental cue variation, and forced perspective shifts). Metacognitive interventions (e.g., reflective prompts, usage dashboards, and goal-setting features) to engage system-2 evaluation might be more effective with satisfaction-driven compulsion (deliberate pathway). In VR-based EPC environments with a high sense of presence and high cognitive satisfaction, patterns of problematic overuse can be observed, particularly with users who have established strong habitual usage patterns.

VR-based EPC consumption typically requires a private setting, and the IGM’s findings suggest that habitual high-presence engagement may progressively isolate users by reinforcing automatic re-engagement cycles. This has implications for digital wellbeing researchers, who should develop VR-specific screening tools that separately measure presence-based and satisfaction-based compulsion dimensions, given their different intervention profiles. Presence-driven compulsion, the automatic, habit-amplified pathway, is best addressed through contextual disruption strategies (mandatory session breaks, cue variation, perspective shifts). Satisfaction-driven compulsion, the deliberate pathway, responds better to metacognitive interventions (usage dashboards, reflective prompts, and goal-setting).

From a policy standpoint, VR content platforms should consider implementing adaptive algorithms that detect escalating usage patterns, particularly increasing session duration and inter-session frequency, as early indicators of automatic pathway activation. Existing age-verification and content restriction frameworks, currently calibrated for 2D EPC, require rethinking for VR environments, where the presence affordance creates qualitatively heightened engagement intensity. The IGM provides a theoretically grounded architecture for designing such platform-level interventions.

For media literacy practitioners, the IGM suggests that awareness-building programs targeting VR-EPC compulsion should address both pathways: not only the hedonic motivations and cognitive satisfactions that drive deliberate engagement but also the automatic presence-triggered cycles that operate below conscious awareness. This dual-pathway literacy approach is more comprehensive than approaches targeting content motivations alone.

### 5.3 Limitations and future studies

This study has several limitations. First, the mono-method quantitative design relies solely on self-report instruments, which are susceptible to social desirability bias, particularly salient for sensitive behaviors such as VR-EPC consumption. Future research should strengthen validity through experiments, VR usage logs, and physiological measures of presence and arousal. As the study used a cross-sectional design, the findings reflect associations rather than causal relationships. Longitudinal and experimental studies are needed to better establish directionality within the IGM framework. In addition, the sample was predominantly male and recruited from Facebook VR-EPC communities, which may overrepresent heavy and habitual users. This selection bias may have amplified observed associations relative to a general VR-user population. Findings should be interpreted as characterizing an active-user subgroup. Future studies should purposively recruit female users, casual consumers, and culturally diverse samples to test the IGM’s boundary conditions. Fourth, the IGM does not explicitly model all relevant VR affordances, interactivity, haptic feedback, avatar embodiment, nor all gratification dimensions such as social presence or emotional regulation. Future research should develop a more comprehensive immersive gratification taxonomy spanning these affordances across content types (EPC, gaming, and social VR) and demographic groups. Additionally, gratification-seeking dispositions were measured as static traits; experience-sampling methodology would better capture the dynamic, episode-by-episode updating of gratifications that U&G theory anticipates. Furthermore, the study did not find statistically significant evidence that habit moderated the VCS → VRA association (H7b: *β* = 0.03, *p* ≥ 0.05). However, a non-significant interaction does not by itself establish the absence of moderation. Future studies should confirm this null pattern using methods specifically designed to evaluate negligible or absent effects, such as equivalence testing (Lakens et al., 2018) or Bayesian approaches that can quantify evidence for the null hypothesis (Altman and Bland, 1995; Dienes, 2014). Aggregate-level structural model outputs are available from the corresponding author upon reasonable request. Item-level raw data are no longer available due to storage limitations.

## 6 Conclusion

This study developed and tested the Immersive Gratification Model (IGM), integrating uses-and-gratifications (U&G) theory with dual-process theory, to explain problematic engagement with erotic/pornographic content in VR environments. Using PLS-SEM with data from 388 adult VR-EPC consumers, we confirmed that three distal gratification-seeking predictors (SC, VSE, and ESEPCP) are each positively associated with two proximal immersive experience evaluations: VR sense of presence (VRP) and VR cognitive satisfaction (VCS), which in turn predict problematic VR-EPC engagement (VRA). Sequential mediation was supported through both the automatic (VRP) and deliberate (VCS) pathways. Crucially, habit asymmetrically moderated these pathways, amplifying VRP → VRA but not the self-sustaining VCS → VRA pathway, confirming the IGM’s central dual-process prediction. The IGM makes three contributions. Theoretically, it extends U&G scholarship to immersive VR by demonstrating that VR-specific gratification architectures create compulsion pathways beyond existing screen-media models. Empirically, it provides the first test of asymmetric dual-process habit moderation in immersive media compulsion. Practically, the pathway-differentiated structure calls for distinct intervention architectures: contextual disruption for automatic presence-driven pathways, and metacognitive reflection tools for deliberate satisfaction-driven pathways. As VR adoption accelerates, theoretically grounded frameworks, such as the IGM, will be essential for advancing immersive media psychology and designing effective digital wellbeing interventions.

## Data Availability

The datasets presented in this study are not publicly available due to storage limitations. Aggregate-level structural model outputs (path coefficients and model fit indices) are available from the corresponding author upon reasonable request.

## Supplementary material

SUPPLEMENTARY TABLE S1 Full item wording for all constructs (SC, VSE, ESEPCP, VRP, VCS, Hbt, VRA) with source scales.

Supplementary Table S2 Common method bias full collinearity VIF results.
