# Supplementary material for: Immersive gratifications and compulsive VR media engagement: a dual-process model of erotic content consumption in virtual reality
Source: Front Psychol. 2026 Jul 2;17:1837501. doi: 10.3389/fpsyg.2026.1837501 (PMC13373093; doi:10.3389/fpsyg.2026.1837501)
Supplement: SUPPLEMENTARY TABLE S1 — Full item wording for all constructs (SC, VSE, ESEPCP, VRP, VCS, Hbt, VRA) with source scales. [file Table_1.DOCX]

**Immersive Gratifications and Compulsive VR Media Engagement: A Dual-Process Model of Erotic Content Consumption in Virtual Reality**

Appendix A

Behavioural studies focusing on ADDICTION

Table 1: Addiction: literature summary.

| **Addiction type** | **Author** | **Findings** |
| --- | --- | --- |
| Addiction SNS | Balcerowska et al. (2020) | SNS Addiction  Facebook Addiction  Comparison of SNS and Facebook Addiction in Poland |
| Addiction technology | Çınar, Bahçeci, & Dikmen (2020) | Internet Addiction among High School Students |
| Addiction games / pleasure | Adams et al., (2018)  Misra, Singh, & Singh (2020) | Anxiety Increase IGD Risk.  Levels of addiction in Active and Non-Active online users are different. |
| Addiction gambling | Brooks, Ferrari, & Clark (2020) | Gambling-related cognitive distortions emerge and Decision-making difficulties. |
| Addiction learning | Perales et al. (2020) | Behavioral Addiction |
| Addiction Mobile | Barnes, Pressey, & Scornavacca, (2018) | Problematic Usage, Users Behavior and addiction |

Appendix B

Virtual Reality: for education, entertainment and commercialization

Virtual reality is an immersive combination of fantasy, immersion and connectivity. These characteristics are considered more efficient when the VR experience involves several sensory channels. Sensorial approaches include vision, sound, sensation, scent and taste (Carulli et al., 2015).Moreover, Chen & Hsu, (2020) stated that VR allows people the chance to live and witness circumstances "with time limitations, physical inaccessibility, specific risks, ethical questions" that cannot be easily reached. Virtual Reality (VR) is becoming a strategic tool for infotainment and entertainment due to its wide market reach and potential for market growth, VR technology is seen as a accessible tool by numerous industries including e-commerce, real estate and sport (Jang and Park, 2019). VR market expected to cross US$ 120 Billion by the year 2020 (Digi-Capital, 2016). Therefore, VR presence is observed to be maximized over the spectrum of Information Communication Technology (ITC) (Cuperus et al., 2017).VR offers an immeasurable virtual environment for the users (Slater and Sanchez-Vives, 2016). Siegle (2019) also suggests that VR helps users to 'take their own participation in situations where they are not present physically but feel comfortable exploring worlds.'.

Although, the first trace of VR witnessed three decades ago. However, it commercially succeeds after Y2K (i.e. Sony-Play station, Google, Oculus Rift and HTC Vive)(Wood et al., 2017). In the recent slice of time, VR based commercial solutions for infotainment and entertainment are getting attention (Ariningsih et al., 2016).Moreover, Literature has extensively studied the use of the technology of VR as a motivating tool for education (Selzer et al., 2019). Virtual reality learning environments’ also arisen in educational establishments as a viable option(Jang et al., 2017). For instance, Merchant et al. (2014) argued that VR enhances the learning ability among youngsters like School and College. Bergin(2015) stated that VR environment supports individuals' cognitive abilities to learn as a VR environment facilitates media richness. Fromberger et al. (2015) highlighted the urge to have open-access freeware VR based medical educational applications as its intensively been in fashion. Researcher highlight that positive learning results may be achieved when incorporating HMDs in realistic settings in preparation with coping competence, the capacity to physically and mentally respond, medical abilities and health competencies in various risky circumstances (Çakiro and Göko, 2019). Similarly, VR has been widely used in education and medical training for years (Southgate, 2018) and it is improving its quality and standard day by day. Similarly, Ineke (2019) conclude VR as an integral part of the future world of entertainment. This is important to note that even as the VR industry has identified different areas such as education and communications, entertainment is still a crucial element in embracing technology given the various intentions. Thus, most customers seem aware of VR, regardless of their real intent, as an entertainment medium (Jung and Lee, 2018).

Appendix C

Table 2: Constructs, instruments, and sources.

| **Construct** | **Code** | **Items description** | **Source** |
| --- | --- | --- | --- |
| Sensual Curiosity (SC) | SC1 | Viewing EPC through VR helps me to learn more about a sexual activity / practice. | (Kim et al., 2018; adapted by Leon-larios et al., 2019) |
|  | SC2 | I’m curious about what types of sex other people have. |  |
|  | SC3 | I use VR to view EPC to expand my knowledge about sexual possibilities. |  |
|  | SC4 | Viewing EPC through VR fuels an interest I have to understand more about sex |  |
| Excitement-Seeking and EPC Pleasure (ESEPCP) | ESEPCP1 | When I watch EPC in VR environment, gives me sense of excitement. | (Kim et al., 2018; adapted by Leon-larios et al., 2019) |
|  | ESEPCP2 | When I watch EPC in VR environment, it helps me to sexually arouse myself |  |
|  | ESEPCP3 | When I watch EPC in VR environment It help me to masturbate and for physical release. |  |
| **Virtual Self-Efficacy (VSE)** | VSE1 | Opening and operating VR based EPC is easy for me. | (Suki and Ramayah, 2010) |
|  | VSE2 | I can control the process of setting up VR gadget to watch EPC. |  |
|  | VSE3 | I have enough time to consumer VR based EPC. |  |
| **VR Presence (VRP)** | VRP1 | The VR based EPC create a new world for me, and the world suddenly disappeared when I finished VR experience. | (Bogicevic et al., 2019) |
|  | VRP2 | The world generated by EPC seemed to me like “something I experienced” rather than “something I watch." |  |
|  | VRP3 | While I was watching EPC, my body was in the room, but my mind was inside the world created by EPC. |  |
| **VR Cognitive Satisfaction (VCS)** | VCS1 | I am delighted to use VR based EPC. | (Kim et al., 2018) |
|  | VCS2 | Using the VR based EPC makes me feel happy. |  |
|  | VCS3 | Using the VR based EPC makes me feel enjoyable. |  |
| Problematic VR-EPC Engagement (VRA) | VRA1 | Using VR based EPC sometimes interferes with other things | (Gong, Yu, & Luqman, 2020) |
|  | VRA2 | When I watch EPC without VR, I often feel agitated. |  |
|  | VRA3 | I have made unsuccessful attempts to reduce the time using VR gadget to watch EPC. |  |
| **Habit (Hbt)** | Hbt1 | Using VR gadget has become a habit. | (Pillet et al., 2016) |
|  | Hbt2 | I use the new VR gadgets automatically |  |
|  | Hbt3 | When I watch EPC, I prefer to use VR gadgets to view EPC. |  |

Appendix D

Table 2: Model fitness indices.

| **Fitness Indices** | **Recommended Value** | **Measurement** | **Proposed** |
| --- | --- | --- | --- |
| **Chi-square** |  | 171.991 | 173.546 |
| **df** |  | 127 | 126 |
| **Chi-square/df** | ≤ 5.0 | 1.354 | 1.377 |
| **GFI** | .95 | .956 | .955 |
| **AGFI** | .90 | .934 | .933 |
| **TLI** | .95 | .986 | .985 |
| **IFI** | .95 | .990 | .989 |
| **NFI** | .95 | .962 | .962 |
| **CFI** | .95 | .990 | .989 |
| **RMSEA** | ≤ .08 | .030 | .031 |
| Note: df= degree of freedom, GFI, AGFI, TLI, NFI, CFI, RMSEA=  Recommended values followed cutoff’s as advised by Hu and Bentler (1999) | | | |

Appendix E

Table 4: Parameter estimation for the proposed model.

| **Sr.** | **Description** | **Beta (β)** | **Significance** | **Result** |
| --- | --- | --- | --- | --- |
| **H1(a)** | SC→VRP | .150 | ≤.01 | Supported |
| **H1(b)** | SC→VCS | .383 | ≤.001 | Supported |
| **H2(a)** | VSE→VRP | .164 | ≤.01 | Supported |
| **H2(b)** | VSE→VCS | .142 | ≤.01 | Supported |
| H3(a) | ESEPCP→VRP | .304 | ≤.001 | Supported |
| H3(b) | ESEPCP→VCS | .470 | ≤.001 | Supported |
| **H4(a)** | VRP→VRA | .221 | ≤.001 | Supported |
| **H4(b)** | VCS→VRA | .286 | ≤.001 | Supported |
| **H7(a)** | VRP×Hbt→VRA | .116 | ≤.05 | Supported |
| **H7(b)** | VCS×Hbt→VRA | .03 | ≥.05 | Theoretical Null retained |

References

Adams, B.L.M., Stavropoulos, V., Burleigh, T.L., Liew, L.W.L., Beard, C.L., Griffiths, M.D., 2018. Internet Gaming Disorder Behaviors in Emergent Adulthood : a Pilot Study Examining the Interplay Between Anxiety and Family Cohesion.

Ariningsih, P.K., Nainggolan, M., Sandy, I., Widyasti, D., 2016. Understanding the Impulsiveness Effect of a Web Design on Online Fashion Stores * 591–597.

Balcerowska, J.M., Bereznowski, P., Biernatowska, A., Atroszko, P.A., Pallesen, S., Andreassen, C.S., 2020. Is it meaningful to distinguish between Facebook addiction and social networking sites addiction ? Psychometric analysis of Facebook addiction and social networking sites addiction scales.

Barnes, S.J., Pressey, A.D., Scornavacca, E., 2018. Mobile Ubiquity : Understanding the Relationship between Cognitive Absorption , Smartphone Addiction and Social Network Services, Computers in Human Behavior. Elsevier B.V. https://doi.org/10.1016/j.chb.2018.09.013

Bergin, R., 2015. Media Richness. Dep. Homel. Def. Secur.

Bogicevic, V., Seo, S., Kandampully, J.A., Liu, S.Q., Rudd, N.A., 2019. Virtual reality presence as a preamble of tourism experience : The role of mental imagery. Tour. Manag. 74, 55–64. https://doi.org/10.1016/j.tourman.2019.02.009

Brooks, G., Ferrari, M., Clark, L., 2020. Cognitive factors in gambling disorder , a behavioral addiction, Cognition and Addiction. Elsevier Inc. https://doi.org/10.1016/B978-0-12-815298-0.00015-0

Çakiro, Ü., Göko, S., 2019. Development of fire safety behavioral skills via virtual reality 133, 56–68. https://doi.org/10.1016/j.compedu.2019.01.014

Carulli, M., Bordegoni, M., Cugini, U., Bordegoni, M., Cugini, U., 2015. Integrating Scents Simulation in Virtual Reality Multisensory Environment for Industrial Products Evaluation Integrating Scents Simulation in Virtual Reality Multisensory Environment for Industrial Products Evaluation. Assoc. Comput. Mach. 4360. https://doi.org/10.1080/16864360.2015.1114390

Chen, Y., Hsu, C., 2020. Self-regulated mobile game-based English learning in a virtual reality environment. Comput. Educ. 103910. https://doi.org/10.1016/j.compedu.2020.103910

Çınar, M., Bahçeci, F., Dikmen, S., 2020. Examining internet addiction levels of high school last-grade students 15, 233–241. https://doi.org/10.5897/ERR2020.3901

Cuperus, A.A., Keizer, A., Evers, A.W.M., Houten, M.M.L. Van Den, Teijink, J.A.W., Ham, I.J.M. Van Der, 2017. Manipulating spatial distance in virtual reality: Effects on treadmill walking performance in patients with intermittent claudication. Comput. Human Behav. https://doi.org/10.1016/j.chb.2017.10.037

Digi-Capital, 2016. Augmented/Virtual Reality revenue forecast revised to hit $120 billion by 2020 [WWW Document]. Augment. Real. Rep. 2016.

Fromberger, P., Meyer, S., Kempf, C., Jordan, K., Müller, J.L., 2015. Virtual viewing time: The relationship between presence and sexual interest in androphilic and gynephilic men. PLoS One 10, 1–28. https://doi.org/10.1371/journal.pone.0127156

Gong, M., Yu, L., & Luqman, A. (2020). Understanding the formation mechanism of mobile social networking site addiction: evidence from WeChat users. *Behaviour & Information Technology*, *39*(11), 1176–1191.

Ham, I.J.M. Van Der, Klaassen, F., Schie, K. Van, Cuperus, A., 2019. Computers in Human Behavior Elapsed time estimates in virtual reality and the physical world : The role of arousal and emotional valence. Comput. Human Behav. 94, 77–81. https://doi.org/10.1016/j.chb.2019.01.005

Hu, L., Bentler, P.M., 1999. Cutoff criteria for fit indexes in covariance structure analysis: Conventional criteria versus new alternatives. Struct. Equ. Model. A Multidiscip. J. 6, 1–55. https://doi.org/10.1080/10705519909540118

Jang, S., Vitale, J.M., Jyung, R.W., Black, J.B., 2017. Direct manipulation is better than passive viewing for learning anatomy in a three- dimensional virtual reality environment. Comput. Educ. https://doi.org/10.1016/j.compedu.2016.12.009

Jang, Y., Park, E., 2019. An adoption model for virtual reality games: The roles of presence and enjoyment. Telemat. Informatics 42, 101239. https://doi.org/10.1016/j.tele.2019.101239

Jung, K.E., Lee, H., 2018. The adoption of virtual reality devices : The technology acceptance model integrating enjoyment , social interaction , and strength of the social ties. Telemat. Informatics. https://doi.org/10.1016/j.tele.2018.12.006

Kim, M., Park, M., Park, J., Kim, J., Kim, E., Kim, M., Park, M., 2018. The role of multidimensional switching barriers on the cognitive and affective satisfaction-loyalty link in mobile communication services: Coupling in moderating effects. Comput. Human Behav. https://doi.org/10.1016/j.chb.2018.05.024

Leon-larios, F., Saavedra-macias, F.J., Albar-, M.J., Gomez-baya, D., 2019. Pornography influence among young students: adaptation and validation of pornography consumption inventory into Spanish. J. Sex Marital Ther. https://doi.org/10.1080/0092623X.2019.1610124

Merchant, Z., Goetz, E.T., Cifuentes, L., Keeney-Kennicutt, W., Davis, T.J., 2014. Effectiveness of virtual reality-based instruction on students’ learning outcomes in K-12 and higher education: A meta-analysis. Comput. Educ. https://doi.org/10.1016/j.compedu.2013.07.033

Misra, R., Singh, S., Singh, N., 2020. Assessing Behavioral Patterns for Online Gaming Addiction: A Study Among Indian Youth 10, 43–64. https://doi.org/10.4018/IJCBPL.2020040104

Perales, J.C., King, D.L., Navas, J.F., Schimmenti, A., Sescousse, G., Starcevic, V., Holst, R.J. Van, Billieux, J., 2020. Neuroscience and Biobehavioral Reviews Learning to lose control : A process-based account of behavioral addiction. Neurosci. Biobehav. Rev. 108, 771–780. https://doi.org/10.1016/j.neubiorev.2019.12.025

Pillet, J., Daniel, K., Carillo, A., 2016. Email-free collaboration : An exploratory study on the formation of new work habits among knowledge workers. Int. J. Inf. Manage. 36, 113–125. https://doi.org/10.1016/j.ijinfomgt.2015.11.001

Selzer, M.N., Gazcon, N.F., Larrea, M.L., 2019. Effects of virtual presence and learning outcome using low-end virtual reality systems ☆. Displays 59, 9–15. https://doi.org/10.1016/j.displa.2019.04.002

Siegle, D., 2019. Seeing Is Believing Using Virtual and Augmented Reality to Enhance Student Learning 46–52. https://doi.org/10.1177/1076217518804854

Slater, M., Sanchez-Vives, M. V., 2016. Enhancing our lives with immersive virtual reality. Front. Robot. AI. https://doi.org/10.3389/frobt.2016.00074

Southgate, A.E., 2018. Immersive virtual reality , children and school education : A literature review for teachers .

Suki, N.M., Ramayah, T., 2010. User Acceptance of the E-Government Services in Malaysia : Structural Equation Modelling Approach. Interdiscip. J. Information, Knowledge, Manag. 5.

Wood, M., Wood, G., Balaam, M., 2017. “They’re just tixel pits, man”: Disputing the ‘Reality’ of virtual reality pornography through the story completion method. Conf. Hum. Factors Comput. Syst. - Proc. 2017-May, 5439–5451. https://doi.org/10.1145/3025453.3025762
